# Unanticipated discovery: basal metabolic rate as an independent risk factor for metabolic dysfunction-associated steatotic liver disease in a 5-year longitudinal cohort study of non-obese individuals in China

**DOI:** 10.3389/fmed.2025.1569655

**Published:** 2025-09-01

**Authors:** Jian Luo, Danfeng Gong, Min Guo, Long Cheng, Xueyan Wu

**Affiliations:** ^1^Department of Endocrinology, Changde Hospital, Xiangya School of Medicine, Central South University, Changde, China; ^2^Department of Gastroenterology, Changde Hospital, Xiangya School of Medicine, Central South University, Changde, China

**Keywords:** basal metabolic rate, metabolic dysfunction-associated fatty liver disease, nonalcoholic fatty liver disease, insulin resistance, mediation analysis

## Abstract

**Objective:**

The role of basal metabolic rate (BMR) in Metabolic Dysfunction-Associated Steatotic Liver Disease (MASLD) remains controversial, with previous studies yielding inconsistent results. The precise relationship remains poorly understood, particularly in non-obese individuals. This study aimed to investigate the longitudinal association between BMR and incident MASLD in a large, non-obese Chinese cohort.

**Methods:**

This longitudinal cohort study included 16,173 non-obese participants free of MASLD at baseline. They were prospectively followed up for 5 years, with the outcome event being the development of MASLD. Participants were divided into quartiles based on their basal metabolic rate (BMR). The association between BMR and incident MASLD was examined using both Cox regression models and restricted cubic spline analysis (RCS).

**Results:**

During the 5-year follow-up period, 2,322 non-obese participants developed MASLD. Multivariate Cox regression analysis revealed that after fully adjusting for relevant confounding factors, the BMR was positively associated with incident MASLD, and the risk of MASLD gradually increased with increasing BMR (HR: 1.3, 95% CI: 1.3, 1.4; *p* for trend < 0.0001). Using RCS regression, we found a positive linear correlation between the BMR and the risk of incident MASLD. Stratified analysis revealed an association between the BMR and increased incidence of MASLD in all the subgroups. Additionally, significant interactions were found between BMR and sex, systolic blood pressure (SBP), uric acid (UA), creatinine (CR), and triglycerides (TGs) (*p* for interaction < 0.05). Mediation analysis indicated that insulin resistance mediated 5.16% of the effect of the BMR on incident MASLD.

**Conclusion:**

In this non-obese Chinese cohort, an elevated BMR was identified as an independent risk factor for incident MASLD. This suggests that BMR could be a valuable early biomarker for MASLD risk stratification, even in individuals without obesity.

## 1 Introduction

Metabolic dysfunction-associated fatty liver disease, formerly known as nonalcoholic fatty liver disease (NAFLD), is a common chronic liver disease. This change in nomenclature reflects a shift toward a diagnosis based on metabolic dysfunction rather than the exclusion of other liver diseases(MASLD) (1). The clinical manifestations of MASLD include hepatic steatosis, steatohepatitis, fibrosis, and cirrhosis. It is significantly associated with the development of hepatocellular carcinoma, which poses a serious threat to human health and increases the social burden (2, 3). Currently, the global prevalence of MASLD is approximately 30%, and its incidence is increasing (4).

Obesity is considered the main risk factor for MASLD (5); however, approximately 20% of MASLD cases occur in non-obese individuals (6–9). Non-obese MASLD, despite the absence of obesity, is not a benign condition. It can progress to severe liver diseases, such as hepatic fibrosis and cirrhosis, highlighting the need for early identification of this high-risk population (10, 11). Compared with healthy individuals, non-obese MASLD patients have a greater incidence of dyslipidemia, arterial hypertension, insulin resistance, and diabetes (12). Researchers have reported that these patients have increased all-cause mortality, cardiovascular mortality, and poor long-term outcomes (13). Among the MASLD population, non-obese patients have similar risks of hepatic decompensation, malignancy, and cardiovascular disease as obese patients do (14). Some researchers have even reported that non-obese MASLD patients have higher fibrosis scores, cardiovascular incidence rates, and late all-cause mortality rates and poorer prognoses than obese MASLD patients do (15).

Since MASLD also poses a threat to the health of non-obese patients, it is particularly important to identify non-obese individuals at risk of MASLD early and take timely intervention measures (16, 17). However, unlike obese patients, non-obese MASLD patients often lack clinical manifestations in the early stages and are therefore frequently overlooked. Currently, liver biopsy is the gold standard for diagnosis; however, owing to the high skill requirements for the operator and the invasive nature of the procedure, it is not widely accepted by patients (18). Ultrasonography, CT, and MRI are good alternative methods, but they are not widely used in rural areas, community hospitals, or large epidemiological surveys in China (18, 19). Therefore, the challenge for the future lies in the early identification of MASLD risk factors through simple, convenient, and noninvasive means (20).

BMR is the sum of energy expenditure by tissues and organs during fasting, in the resting state, and under thermoneutral conditions (21). Obesity and metabolic disorders result from a mismatch between energy intake and expenditure (22). Thus, abnormalities in BMR may lead to metabolic disorders, which in turn can cause insulin resistance (IR), dyslipidemia, and other conditions (23, 24), all of which are important factors contributing to the development of MASLD (25, 26). The relationship between BMR and MASLD remains ambiguous, with current studies presenting conflicting findings (27–29). A primary reason for this uncertainty is the paucity of large-scale, prospective cohort studies designed to establish a clear longitudinal association. Therefore, the present study leverages a large, community-based cohort to prospectively evaluate the independent link between BMR and the incidence of MASLD.

## 2 Materials and methods

### 2.1 Study design and participants

This was a longitudinal cohort study with original data sourced from a public database.^1^ In accordance with Dryad’s terms of service, the data in this database are available for free use by researchers (30). A total of 16,173 non-obese patients without MASLD at baseline were included in the study (30). The exclusion criteria at baseline were as follows: (1) incomplete clinical data and failed follow-up; (2) currently taking oral antihypertensive, lipid-lowering, or antidiabetic medications; (3) excessive alcohol consumption (140 g/w for males, 70 g/w for females); (4) coexisting MASLD, autoimmune hepatitis, viral hepatitis, or chronic liver disease of known etiology; (5) LDL-C > 3.12 mmol/L; and (6) body mass index (BMI) ≥ 25 kg/m^2^. All participants provided informed consent, and the study protocol was approved by the Ethics Committee of Wenzhou People’s Hospital. Our study was a secondary analysis of a publicly available dataset. The original study protocol was approved by the Ethics Committee of Wenzhou People’s Hospital, and all participants had provided informed consent.

### 2.2 Data collection

Medical history and lifestyle habits were collected via questionnaires administered by trained physicians. The participants were seated in a quiet environment, and their blood pressure was measured via an automatic sphygmomanometer. Standing height and weight were measured without shoes and with the participants in light clothing. Blood samples were collected in the morning after an overnight fast to assess biochemical indicators, including alanine aminotransferase (ALT), aspartate aminotransferase (AST), gamma-glutamyl transpeptidase (GGT), alkaline phosphatase (ALP), total bilirubin (TB), albumin (ALB), globulin (GLB), creatinine (Cr), urea nitrogen (BUN), fasting plasma glucose (FPG), uric acid (UA), total cholesterol (TC), triglyceride (TG), high-density lipoprotein cholesterol (HDL-C), and low-density lipoprotein cholesterol (LDL-C) levels.

### 2.3 Definitions and outcome variables

BMR was calculated via the Harris–Benedict equation, which is currently one of the better equations for estimating energy expenditure in subjects (31): BMR (male) = 88.362 + (13.397 × weight) + (4.799 × height) - (5.677 × age); BMR (female) = 447.593 + (9.247 × weight) + (3.098 × height) - (4.33 × age). BMI (kg/m^2^) was calculated as the baseline weight (kg) divided by the square of height (m^2^). The metabolic score for insulin resistance [(METS-IR) represents IR] (32): METS-IR = Ln((2 × FPG) + TG) × BMI)/(Ln(HDL-C).

The outcome variable was the incidence of MASLD, which was diagnosed via abdominal ultrasound according to the recommended standards of the Chinese Association for the Study of Liver Diseases (33). MASLD is generally defined as the presence of at least two out of three abnormal findings on abdominal ultrasound examination: (1) diffuse increase in near-field ultrasound echo of the liver (“bright liver”); (2) liver echo greater than the kidney echo; and (3) vascular blurring and progressive attenuation of far-field ultrasound echo.

### 2.4 Statistical analysis

To assess the relationship between the BMR and the incidence of MASLD, participants were grouped on the basis of BMR quartiles (Q1: 1137.8--1225.3; Q2: 1277.0--1321.0; Q3: 1369.2--1425.0; and Q4: 1501.4--1636.2). Given the large values of BMR, we performed a z score transformation on BMR for analysis. For variables with more than 20% missing data, multiple imputation was conducted via the random forest method. Continuous variables are presented as the means ± standard deviations (SDs), whereas categorical variables are presented as medians (Q1--Q3). One-way ANOVA or nonparametric tests were used for continuous variables, and the chi-square test was used for categorical variables.

The cumulative risk of MASLD during follow-up was calculated via Kaplan–Meier analysis. The Cox proportional hazards regression model was used to determine the hazard ratio (HR) and evaluate the association between the BMR z score and the incidence of newly diagnosed MASLD across six models (crude model adjusted for none; Model 1 adjusted for SEX; Model 2 adjusted for Model 1 plus liver function markers (ALP, GGT, ALT, AST, TP, ALB, GLB, TB, DBIL); Model 3 adjusted for Model 2 plus renal function markers (BUN, CR, UA); Model 4 adjusted for Model 3 plus lipid profile markers (TC, TG, HDL-C, LDL-C); Model 5 adjusted for Model 4 plus FPG and blood pressure). We compared the dose–response relationship between BMR and MASLD using a Cox model with restricted cubic splines (RCSs).

The quartiles of BMR z score were used as continuous variables, with the lowest quartile of BMR used as the reference, and a linear trend test was conducted. Additionally, we performed subgroup analyses to examine the relationship between BMR z score and incident MASLD through potential effect modifiers, and interaction tests were conducted. To assess the mediating role of IR between BMR and MASLD, we conducted a post mediation analysis. The statistical analyses in this study were performed via R version 4.2.0 and Empower Stats version 4.0. Two-tailed *p* values of less than 0.05 were considered to indicate statistical significance.

## 3 Results

### 3.1 Baseline characteristics of the participants

This study included 16,173 participants with a baseline mean age of 43.2 ± 15.0 years. There were slightly more male participants (8,483, accounting for 52.45%) than female participants (7,690, accounting for 47.55%). Table 1 summarizes the baseline characteristics grouped by BMR quartiles. Overall, the BMR of males was greater than that of females. In the high-BMR group (Q4), the AGE, GLB, and HDL-C levels were lower than those in the low-BMR group (Q1). Moreover, as the BMR increased, the levels of ALP, GGT, ALT, AST, TP, ALB, TB, DBIL, BUN, CR, UA, LDL-C, fasting glucose, height, weight, BMI, and blood pressure also increased, with statistically significant differences. There was no statistically significant difference in TC between the high BMR group (Q4) and the low BMR group (Q1).

**Table 1:** Characteristics of the subjects.

BMR quartile	Q1	Q2	Q3	Q4	*p*-value
*N* (%)	4,043	4,043	4,043	4,044	
Sex					<0.001
Female	2,374 (58.7%)	2,585 (63.9%)	2005 (49.6%)	726 (18.0%)	
Male	1,669 (41.3%)	1,458 (36.1%)	2038 (50.4%)	3,318 (82.0%)	
Age, years	54.2 ± 15.6	42.4 ± 14.1	40.0 ± 12.9	36.3 ± 10.4	<0.001
MASLD					<0.001
No	3,803 (94.1%)	3,638 (90.0%)	3,383 (83.7%)	3,027 (74.9%)	
Yes	240 (5.9%)	405 (10.0%)	660 (16.3%)	1,017 (25.1%)	
ALP, U/L	70.8 ± 21.5	71.3 ± 19.8	72.7 ± 19.4	74.6 ± 19.5	<0.001
GGT, U/L	22.0 (17.0–22.0)	22.0 (17.0–22.0)	22.0 (18.0–29.0)	25.0 (20.0–38.0)	<0.001
ALT, U/L	16.0 (13.0–16.0)	16.0 (13.0–17.0)	16.0 (14.0–21.0)	19.0 (15.0–26.0)	<0.001
AST, U/L	22.6 ± 8.3	22.7 ± 8.3	23.2 ± 8.2	23.7 ± 8.2	<0.001
TP, g/L	73.9 ± 4.2	73.7 ± 4.4	73.9 ± 4.1	74.1 ± 4.1	0.005
ALB, g/L	44.0 ± 2.7	44.0 ± 2.7	44.4 ± 2.7	45.2 ± 2.6	<0.001
GLB, g/L	29.9 ± 3.8	29.7 ± 3.9	29.4 ± 4.0	28.9 ± 3.8	<0.001
TB, μmo/L	11.5 ± 3.5	11.8 ± 3.6	12.3 ± 4.1	12.8 ± 4.5	<0.001
DBIL, μmo/L	2.3 ± 0.9	2.3 ± 0.9	2.3 ± 0.9	2.3 ± 1.0	0.017
BUN, mmol/L	4.5 ± 1.5	4.5 ± 1.3	4.6 ± 1.4	4.6 ± 1.2	<0.001
CR, μmo/L	70.2 ± 23.7	73.2 ± 19.6	81.7 ± 33.9	88.8 ± 18.2	<0.001
UA, mmol/L	241.5 ± 72.6	257.2 ± 78.7	291.0 ± 84.7	329.5 ± 79.5	<0.001
FPG, mmol/L	5.1 ± 0.7	5.1 ± 0.8	5.2 ± 0.8	5.2 ± 0.8	<0.001
TC, mmol/L	4.6 ± 0.8	4.6 ± 0.7	4.6 ± 0.8	4.6 ± 0.7	0.094
TG, mmol/L	1.1 ± 0.7	1.2 ± 0.7	1.4 ± 0.9	1.6 ± 1.2	<0.001
HDL-C, mmol/L	1.6 ± 0.4	1.5 ± 0.4	1.4 ± 0.3	1.4 ± 0.3	<0.001
LDL-C, mmol/L	2.2 ± 0.5	2.2 ± 0.5	2.3 ± 0.5	2.3 ± 0.4	<0.001
Height, cm	157.5 ± 5.4	161.9 ± 5.6	166.4 ± 5.6	172.3 ± 5.5	<0.001
Weight, kg	49.7 ± 5.1	54.9 ± 5.2	60.2 ± 5.6	67.5 ± 6.0	<0.001
BMI, kg/m^2^	20.1 ± 1.9	21.0 ± 1.9	21.8 ± 1.8	22.7 ± 1.6	<0.001
SBP, mmHg	117.7 ± 17.7	118.5 ± 16.8	122.1 ± 16.6	124.7 ± 14.6	<0.001
DBP, mmHg	70.0 ± 9.8	71.5 ± 10.2	73.9 ± 10.3	75.9 ± 10.1	<0.001
BMR	1173.2 ± 67.0	1298.8 ± 25.9	1397.6 ± 32.9	1578.4 ± 94.0	<0.001

Values are *n* (%) or mean (standard deviation) or median (Q1-Q3). BMR, Basal metabolic rate; MASLD, Metabolic Dysfunction-Associated Steatotic Liver Disease; ALP, Alkaline phosphatase; GGT, Gamma-glutamyl transferase; ALT, Alanine aminotransferase; AST, Aspartate aminotransferase; TP, Total Protein; ALB, Albumin; GLB, Globulin; TB, Total bilirubin; DBIL, Direct bilirubin; BUN, Blood urea nitrogen; Cr, Creatinine; UA, Uric acid; FPG, Fasting plasma glucose; TC, Total cholesterol; TG, Triglyceride; HDL-C, High-density lipoprotein cholesterol; LDL-C, Low-density lipoprotein cholesterol; BMI, Body mass index; DBP, Diastolic blood pressure; SBP, Systolic blood pressure.

### 3.2 Prevalence of MASLD in the non-obese population

**Figure 1:**
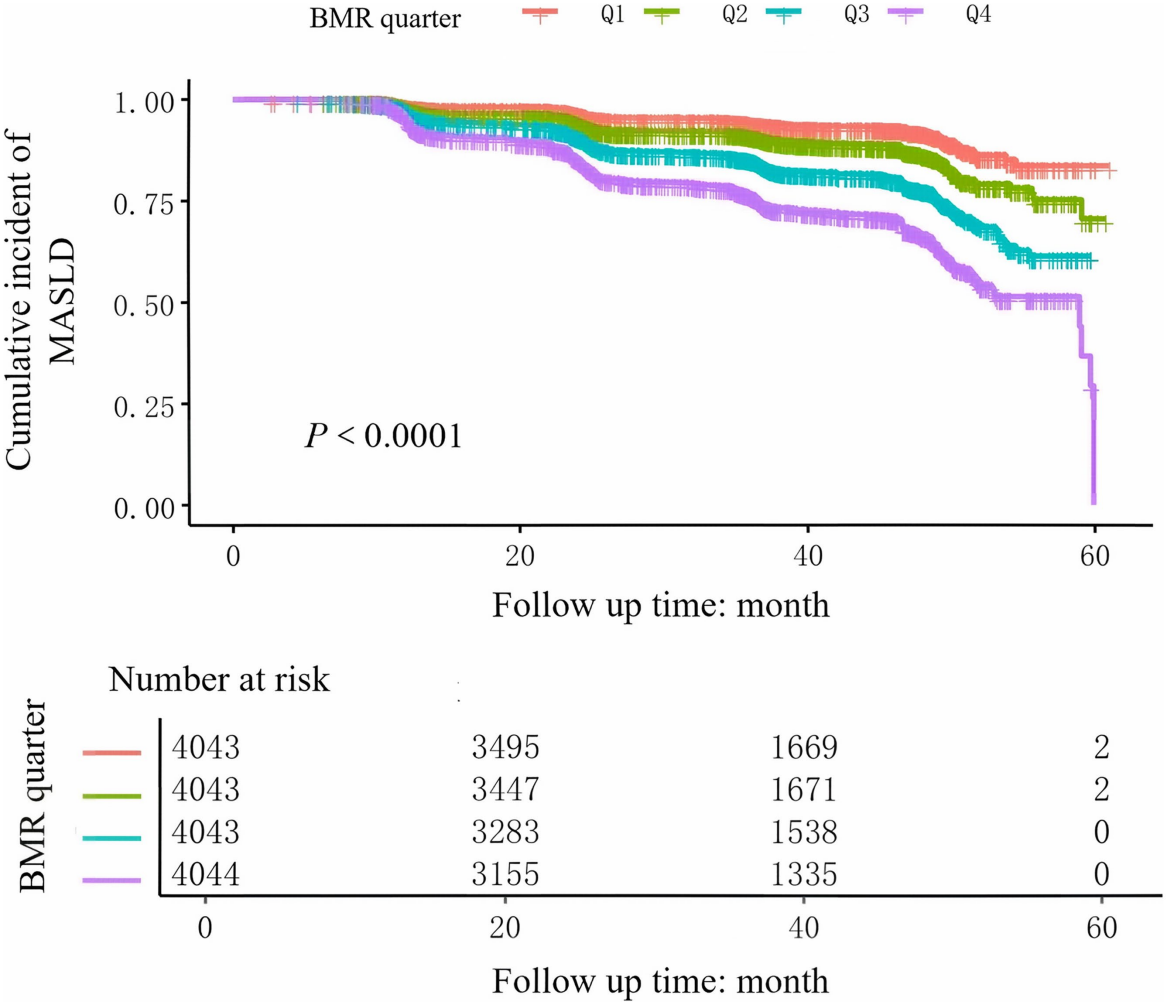
Subgroup analyses for the associations between BMR and MASLD were adjusted for sex, age, SBP, DBP, GGT, ALT, AST, UA, CR, TG, HDL-C, TC, and FPG, with the exception of the stratified variable.

During the 5-year follow-up, 2,322 non-obese participants developed MASLD, with an incidence rate of 14.36%. As shown in Table 1, the prevalence rates of MASLD corresponding to BMI quartiles were as follows: Q1: 5.9%, Q2: 10.0%, Q3: 16.3%, and Q4: 25.1%. The incidence of MASLD gradually increased with increasing BMR. Furthermore, as estimated by the Kaplan–Meier curves (Figure 1), the 5-year cumulative incidence of MASLD events in the four BMR groups increased with increasing BMR (log-rank test *p* < 0.001).

### 3.3 Relationship between the BMR and MASLD

**Figure 2:**
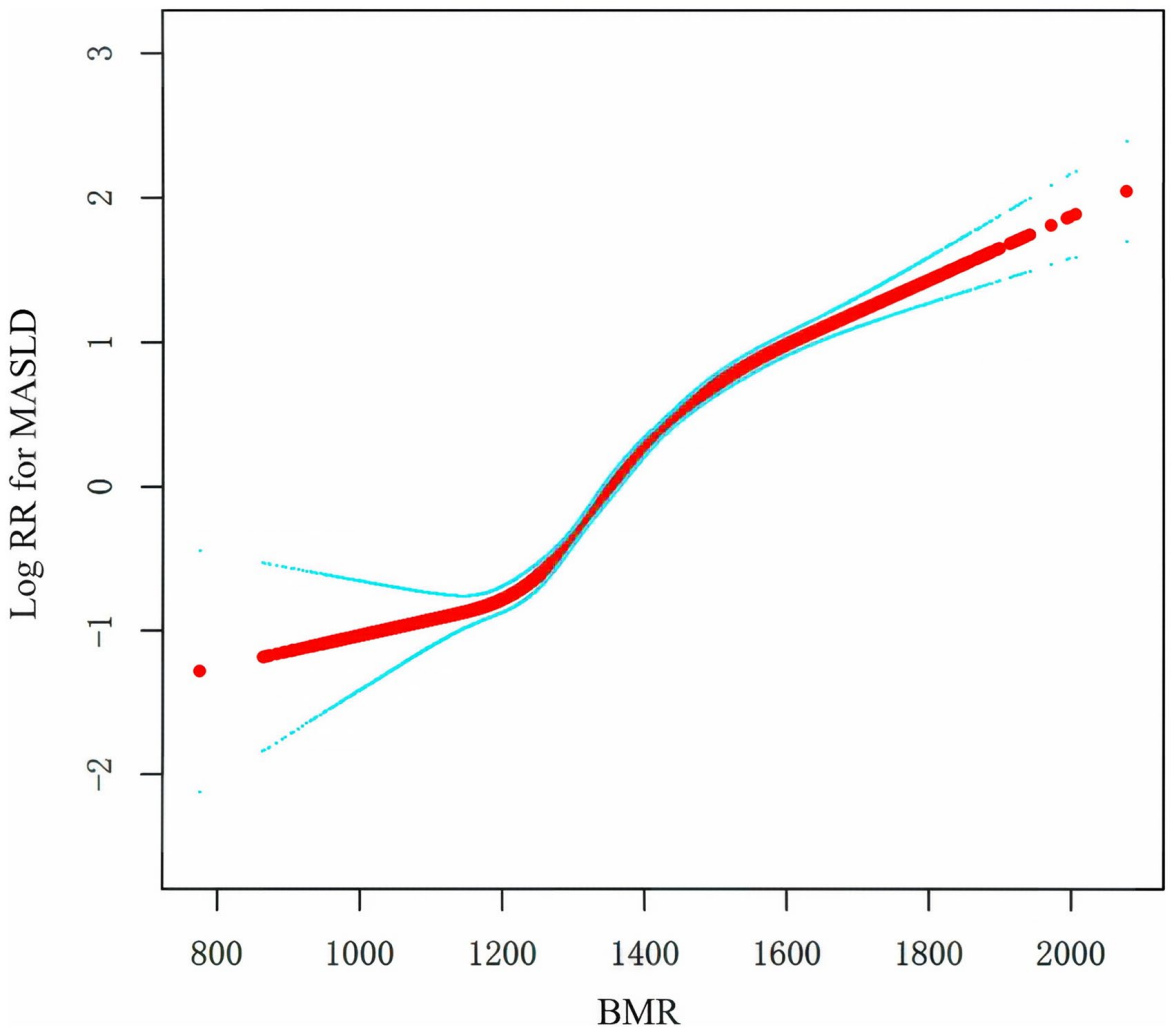
Kaplan–Meier curves of the cumulative incidence of new-onset MASLD stratified by BMR quartile.

To assess the relationship between BMR and MASLD, we constructed multiple models via Cox regression analysis. The results, as shown in Table 2, indicate that in the unadjusted model, the BMR was positively correlated with the risk of MASLD (HR: 1.7, 95% CI: 1.7, 1.8; *p* < 0.001). As the number of adjustment variables increased, the correlation between the two further decreased, but the positive trend remained unchanged. According to the fully adjusted model for confounding variables, for every one-unit increase in BMR-z score, the risk of MASLD increased by 30% (HR: 1.3, 95% CI: 1.3, 1.4; *p* < 0.0001). We further grouped BMR z scores into quartiles and conducted a trend test, with Q1 as the reference. The risk of developing MASLD significantly increased with increasing BMR (*p* for trend < 0.001). In the Cox model with RCS, BMR was also determined to be linearly positively correlated with MASLD (Figure 2).

**Table 2:** Association between BMR and MASLD in different models.

Exposure	MASLD, HR (95%CI)					
	Crude model	model 1	model 2	Model 3	Model 4	model 5
BMR z-score	1.7 (1.7, 1.8) <0.001	1.9 (1.8, 1.9) <0.001	1.6 (1.6, 1.7) <0.001	1.5 (1.4, 1.5) <0.001	1.4 (1.3, 1.5) <0.001	1.3 (1.3, 1.4) <0.001
BMR z-score quartile						
Q1	1.0	1.0	1.0	1.0	1.0	1.0
Q2	1.7 (1.4, 2.0) <0.001	1.7 (1.4, 2.0) <0.001	1.5 (1.3, 1.8) <0.001	1.4 (1.2, 1.7) <0.001	1.4 (1.2, 1.6) <0.001	1.4 (1.1, 1.6) <0.001
Q3	2.9 (2.5, 3.4) <0.001	3.0 (2.6, 3.4) <0.001	2.5 (2.1, 2.9) <0.001	2.0 (1.7, 2.4) <0.001	1.9 (1.6, 2.2) <0.001	1.7 (1.5, 2.0) <0.001
Q4	4.8 (4.2, 5.5) <0.001	5.3 (4.6, 6.2) <0.001	3.9 (3.3, 4.5) <0.001	2.8 (2.4, 3.3) <0.001	2.4 (2.1, 2.9) <0.001	2.1 (1.8, 2.5) <0.001

Crude model adjusted for none. Model 1 adjusted for: SEX; Model 2 adjusted for: SEX; ALP; GGT; ALT; AST; TP; ALB; GLB; TB; DBIL. Model 3 adjusted for: SEX; ALP; GGT; ALT; AST; TP; ALB; GLB; TB; DBIL; BUN; CR; UA. Model 4 adjusted for: SEX; ALP; GGT; ALT; AST; TP; ALB; GLB; TB; DBIL; BUN; CR; UA; TC; TG; HDL-C; LDL-C. Model 5 adjusted for: SEX; ALP; GGT; ALT; AST; TP; ALB; GLB; TB; DBIL; BUN; CR; UA; TC; TG; HDL-C; LDL-C; FPG; SBP; DBP.

### 3.4 Subgroup analysis

**Figure 3:**
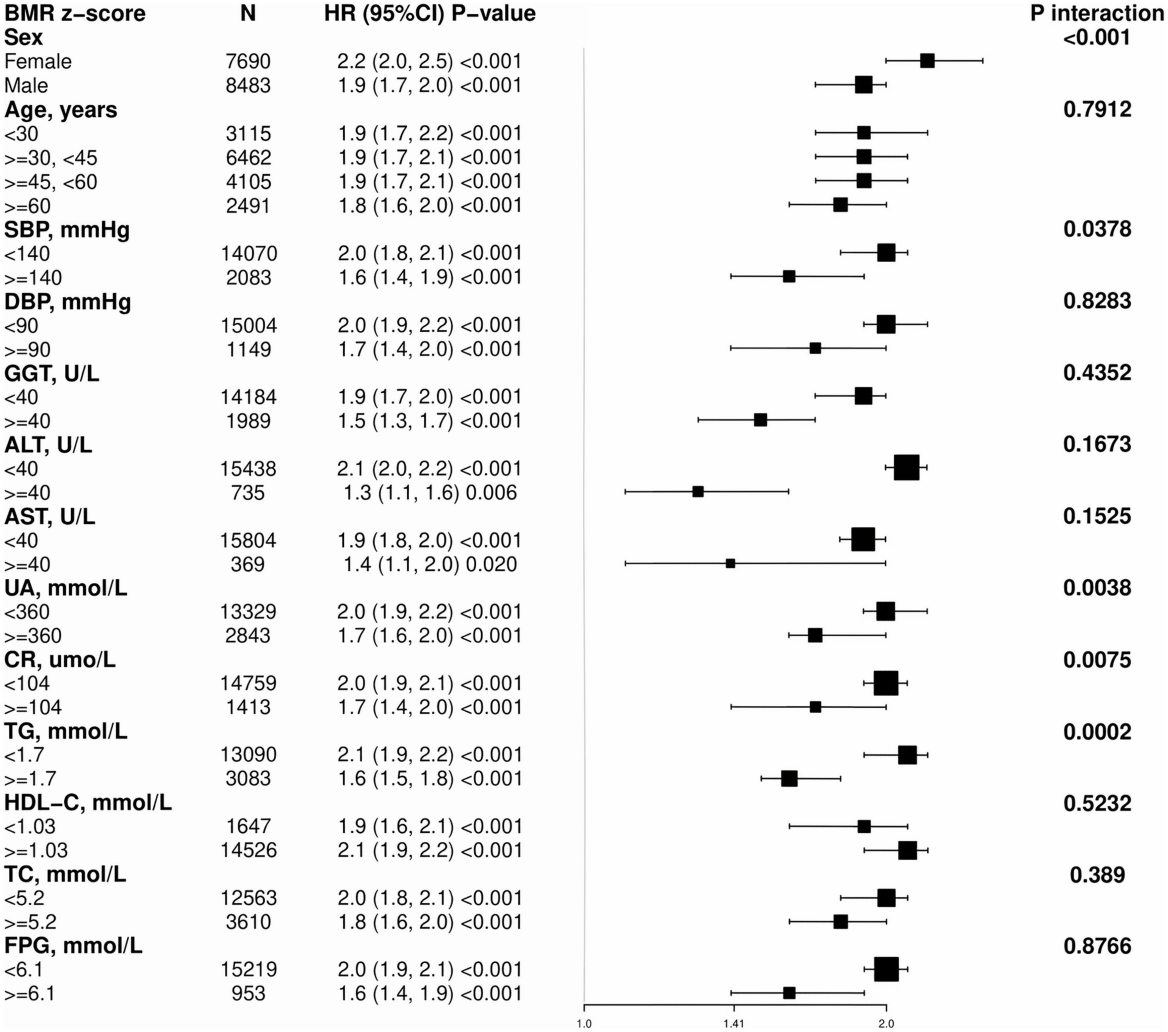
Mediation analysis of the association between the BMR and MASLD.

To further understand the relationship between BMR and MASLD in various subgroups, we used Cox regression models to analyze each stratified group (adjusting for other stratifying variables except for the stratifying variable itself). We employed the likelihood ratio test to examine differences among the stratified groups and determine whether there were interactions (Figure 3). A positive correlation was found between BMR z score and the incidence of MASLD in all the subgroups, and all the correlations were statistically significant. The interaction test revealed that there were interactions for sex, SBP, UA, CR, and TG (interaction *p* < 0.05), whereas no interactions were observed in the subgroups for age, DBP, GGT, AST, HDL-C, TC, or FPG (interaction *p* > 0.05).

### 3.5 Mediation analysis

**Figure 4:**
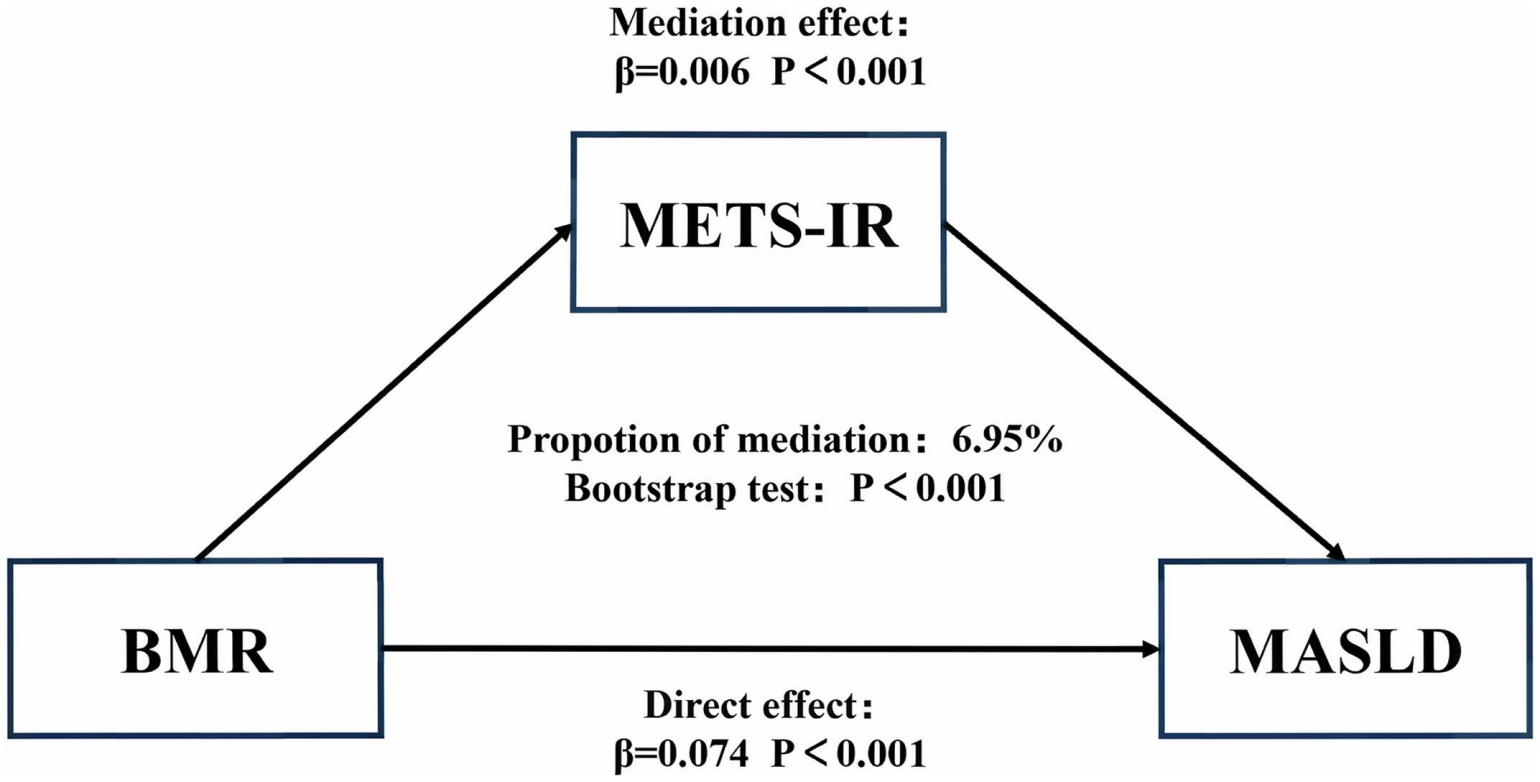
Association of the BMR with the risk of MASLD. Positive relationship between the BMR and MASLD incidence rate. A linear relationship was detected after adjusting for SEX.

To assess the mediating role of IR in the relationship between BMR and MASLD, we conducted a mediation analysis. The results suggested that 6.95% of the relationship between BMR and incident MASLD was mediated by IR (*p* < 0.0001; Figure 4).

## 4 Discussion

In this 5-year longitudinal cohort study of non-obese Chinese individuals, we investigated the association between basal metabolic rate (BMR) and incident metabolic dysfunction-associated steatotic liver disease (MASLD). After adjusting for key confounding factors in a multivariate Cox regression model, we found that elevated BMR was an independent predictor of MASLD (HR: 1.3, 95% CI: 1.3–1.4; *p* for trend < 0.001). Our study is among the first to systematically demonstrate a positive longitudinal association between BMR and MASLD risk in a non-obese Chinese population, offering a new perspective on the risk factors for this condition.

MR refers to the minimum energy expenditure required to maintain life activities at rest, and it is influenced by various factors, including age, sex, muscle mass, endocrine status, and genetics (34). It has previously been used to assess metabolic-related diseases such as hyperthyroidism, hypothyroidism, and diabetes (35). However, with a deeper understanding of BMR, researchers have reported that BMR is a risk factor for various diseases. A large-scale study (*n* = 534,045) using Mendelian randomization analysis revealed an association between BMR and cardiometabolic risk factors (36). Similarly, Li reported that BMR is associated with an increased risk of aortic aneurysm and atrial fibrillation/flutter (37). A higher BMR increases the risk of cancer, and although the specific mechanisms are not yet clear, BMR, as a potential modifiable target for cancer prevention, warrants further investigation (38, 39). Some researchers have even reported that BMR is related to lifespan (40). The onset and progression of MASLD are closely related to energy metabolism disorders, and BMR accounts for 60–70% of total daily energy expenditure (41), playing a crucial role in energy balance. Therefore, we hypothesize that there is a connection between BMR and MASLD (42).

However, previous studies on the relationship between BMR and MASLD have shown conflicting results; researchers have reported that a decrease in BMR leads to reduced caloric consumption, which in turn results in fat accumulation, dyslipidemia, insulin resistance, and obesity (43–46), thereby triggering the onset of MASLD events (47).

Nevertheless, some studies have shown that BMR of individuals with fatty liver is significantly greater than that of healthy individuals (27, 48); moreover, researchers have also reported that BMR of non-obese and obese MASLD patients is similar but significantly greater than that of patients without fatty liver (49). This is consistent with our research findings. Through a larger sample size, we demonstrated a positive correlation between BMR and the incidence of MASLD. Furthermore, through longitudinal follow-up, we established an independent association between them.

The positive association between a high BMR and incident MASLD in a non-obese population is a paradoxical finding that warrants detailed explanation. We speculate on several interconnected pathways.

First, the role of body composition is critical. BMR is determined by the metabolic activity of various tissues, with organs, muscle, and adipose tissue being major contributors. Whether increased muscle mass could explain the higher BMR in our non-obese cohort. While we lacked direct measures of muscle and fat mass, it is plausible that some individuals classified as non-obese by BMI may possess higher muscle mass, which would elevate their BMR. While Table 1 indicates that BMR increases with BMI, a more likely explanation in the context of MASLD is the corresponding increase in adipose tissue, which is metabolically active. A higher BMR could be a marker of “metabolically active” adipose tissue, reflecting increased *de novo* lipogenesis, inflammation, and adipokine secretion, all of which are pathogenic drivers of MASLD. This aligns with the concept of “metabolically obese normal weight” individuals, who have excess visceral adiposity despite a normal BMI.

Second, a high BMR may be a marker of underlying IR: Patients with diabetes have a relatively high BMR due to abnormal protein metabolism and insulin resistance (50). In this study, we used the calculated METS-IR as a representative of IR (32), and the mediation analysis results indicated that 6.95% of the effect of BMR on the incidence of MASLD is mediated by IR. Admittedly, while IR plays a role, the vast majority of this association is likely attributable to other, more direct pathophysiological mechanisms or unmeasured mediators. For instance, factors such as genetic predispositions related to hepatic lipid metabolism, subclinical inflammation, and hormonal status may play a more pivotal role and warrant further investigation.

Third, dyslipidemia and abnormal fat distribution: A higher BMR may lead to increased energy consumption. If energy intake is not balanced, it may trigger the utilization of triglycerides (TGs) stored in adipose tissue as an energy source, which could result in elevated levels of circulating TG, particularly in individuals with sustained increases in BMR (36), thereby triggering the onset of MASLD. Studies have shown that patients with fatty liver have an elevated BMR, and similarly, their visceral fat area is also greater than that of nonfatty liver patients (48). These findings suggest that long-term energy excess can lead to the accumulation of visceral and hepatic fat, thereby promoting the development of MASLD. Notably, individuals who are classified as non-obese on the basis of BMI may still have abdominal obesity.

In the stratified analysis, we found that BMR was a risk factor for incident MASLD (*p* < 0.05) across all subgroups stratified by various indicators, suggesting the robustness of the results. Interestingly, the correlation between BMR and MASLD was stronger in female participants and in the normal population with SBP < 140 mmHg, UA < 360 μmol/L, CR < 104 μmol/L, and TG < 1.7 mmol/L. The stronger association between BMR and MASLD in females is a noteworthy finding. Several factors may explain this gender disparity. First, body composition differs significantly between sexes, with females generally having a higher percentage of body fat and lower muscle mass than males (51). Since adipose tissue is metabolically active and contributes to BMR, differences in fat distribution and metabolism could play a role. Second, sex hormones, particularly estrogen, have a profound impact on energy metabolism and fat deposition. Fluctuations in estrogen levels throughout a woman’s life, especially after menopause, can lead to increased visceral adiposity and insulin resistance, potentially amplifying the effect of a high BMR on MASLD risk (52). Further research is needed to elucidate the precise mechanisms underlying this gender difference. This finding suggests that even individuals with normal baseline test indicators are at risk of developing MASLD, and it is still important to pay attention to BMR and monitor overall metabolic status.

In synthesis, our findings suggest a multifactorial pathway linking elevated BMR to MASLD development in non-obese individuals. A persistently high BMR may signify underlying metabolic inefficiency. This state could foster insulin resistance, a key mediator identified in our analysis, which in turn promotes hepatic *de novo* lipogenesis. Concurrently, a high metabolic rate may increase the mobilization of fatty acids from adipose tissue to meet energy demands, leading to dyslipidemia and ectopic fat accumulation in the liver. These processes, potentially exacerbated by subclinical inflammation and genetic predispositions, collectively create a pro-steatotic environment, even in the absence of obesity. While we propose plausible biological mechanisms, they remain speculative as they were not directly tested in this study.

This study has the following unique advantages: (a) This is the first study to explore the correlation between BMR and incident MASLD in non-obese patients. (b) This was a population-based longitudinal cohort study with a large sample size, reasonable adjustment for statistical covariates, and rigorous statistical analysis, which can elucidate the independent association between BMR and incident MASLD. (c) After rigorous statistical adjustment and sensitivity analysis, the positive correlation between BMR and MASLD remained stable, indicating the reliability of the study conclusions. (d) BMR measurement is almost cost-free and clinically very simple and convenient, making it widely applicable in rural areas, community hospitals, and large epidemiological surveys.

Despite its many advantages, this study also has certain limitations. First, despite adjusting for a comprehensive set of biochemical markers, we could not account for other important potential confounders. Specifically, data on key lifestyle factors (such as dietary intake, physical activity, and sleep quality), genetic predispositions, and direct measures of adiposity like visceral fat area were not collected. The omission of these variables means that residual confounding cannot be fully excluded, which may have introduced bias into the observed associations. Second, the diagnosis of fatty liver was made via ultrasound examination rather than histological examination of course, in clinical practice, ultrasound is the most common noninvasive method for diagnosing MASLD (53). The estimation of BMR using the Harris-Benedict equation rather than direct measurement by indirect calorimetry. While widely used in large-scale epidemiological studies (54), this formula does not account for individual variability in factors such as body composition (e.g., fat mass vs. lean mass) or underlying inflammatory status, which could independently influence metabolic rate and potentially affect the precision of our findings. Additionally, due to data limitations, the underlying mechanisms were not explored. Finally, since the study population consisted of Chinese individuals, considering that metabolic rates may differ among different ethnic groups (55), whether the conclusions of this study are applicable to other populations requires further validation through more cohort studies.

Future research can delve deeper into the underlying mechanisms between BMR and MASLD, such as examining oxidative stress indicators and insulin resistance levels. We can also explore the impact of exercise and diet on the relationship between BMR and MASLD, providing evidence for the prevention and treatment of MASLD.

## 5 Conclusion

In this large-scale longitudinal study of non-obese Chinese individuals, we demonstrate for the first time that an elevated BMR is an independent risk factor for incident MASLD. From a clinical perspective, this finding is of significant importance. It suggests that BMR, a simple and cost-effective measurement, could serve as a novel and easily accessible marker to identify non-obese individuals at high risk for developing MASLD, who might otherwise be overlooked in routine screening. Clinicians should be aware that a high BMR, even in a non-obese individual, may signal underlying metabolic dysregulation. This highlights the need to look beyond BMI in risk stratification and encourages a more comprehensive metabolic assessment. Monitoring BMR could become a practical component of preventative strategies, prompting earlier lifestyle interventions, such as dietary modifications and increased physical activity, to mitigate the risk of MASLD progression in this vulnerable population.

## Data Availability

Publicly available datasets were analyzed in this study. This data can be found at: https://doi.org/10.5061/dryad.1n6c4 from reference (30).

## Footnotes

^1^ https://doi.org/10.5061/dryad.1n6c4

## Glossary

BMR: Basal metabolic rate
MASLD: Metabolic Dysfunction-Associated Steatotic Liver Disease
ALP: Alkaline phosphatase
GGT: Gamma-glutamyl transferase
ALT: Alanine aminotransferase
AST: Aspartate aminotransferase
TP: Total protein
ALB: Albumin
GLB: Globulin
TB: Total bilirubin
DBIL: Direct bilirubin
BUN: Blood urea nitrogen
Cr: Creatinine
UA: Uric acid
FPG: Fasting plasma glucose
TC: Total cholesterol
TG: Triglyceride
HDL-C: High-density lipoprotein cholesterol
LDL-C: Low-density lipoprotein cholesterol
BMI: Body mass index
DBP: Diastolic blood pressure
SBP: Systolic blood pressure
